# Supplementary material for: Heat adaptation of phage T7 under an extended genetic code
Source: Virus Evol. 2021 Dec 1;7(2):veab100. doi: 10.1093/ve/veab100 (PMC8923235; doi:10.1093/ve/veab100)
Supplement: veab100_Supp [file veab100_supp.zip › Sequencing_Analysis.zip!/Sequencing_Analysis/S3/evidence/JC_48.html]

BRESEQ :: Evidence


| New junction evidence | | | | | | | | | | | |
| --- | --- | --- | --- | --- | --- | --- | --- | --- | --- | --- | --- |
|  | | seq id | position | reads (cov) | reads (cov) | score | skew | freq | annotation | gene | product |
| \* | ? | NC\_001604.1 | = 8335 | 1786 (1.040) | 7 (0.000) | 6/584 | 44.4 | 0.39% | intergenic (–/–) | *–/–* | –/– |
|  | ? | NC\_001604.1 | 8939 = | 1866 (1.110) |  |  |  |  | intergenic (–/–) | *–/–* | –/– |
| Rejected: Coverage evenness skew score above cutoff. | | | | | | | | | | | |
| Rejected: Frequency below/above cutoff threshold. | | | | | | | | | | | |

`GGCACCGAGCGTCCACATCATTATCACCCGTGGTGACTTTGAGAAAGCAATAGACAAGAAACGTCCGGTTCTTAGTGTGGCAGTGACCCGCTTCCCGTTCGTCCGTCTGTTACTCAAACGAATCAAGGAGGTGTTCTGATGGGACTGTTAGATGGTGAAGCCTGGGAAAAAGAAAACCCGCCAGTACAAGCAACTGGGTGTATAGCTTGCTTAGAGAAAGATGACCGTTATCCACACACCTGTAACAAAGGAGCTAACGATATGACCGAACGTGAACAAGAGATGATCATTAAGTTGATAGACAATAA‑‑‑‑‑‑‑‑‑‑‑‑‑‑‑‑‑‑‑‑‑‑‑‑‑‑‑‑‑‑‑‑‑‑‑‑‑‑‑‑‑‑‑‑‑‑‑‑‑‑‑‑‑‑‑‑‑‑‑‑‑‑‑‑‑‑‑‑‑‑‑‑‑‑‑‑‑‑‑‑‑‑‑‑‑‑‑‑‑‑‑‑‑‑‑‑‑‑‑‑‑‑‑‑‑‑‑‑‑‑‑‑‑‑‑‑‑‑‑‑‑‑‑‑‑‑‑‑‑‑‑‑‑‑‑‑‑‑‑‑‑‑‑‑‑‑‑‑‑‑‑‑‑‑‑‑‑‑‑‑‑‑‑‑‑‑‑‑‑‑‑‑‑‑‑‑‑‑‑‑‑‑‑‑‑‑‑‑‑‑‑‑‑‑‑‑‑‑‑‑‑‑‑‑‑‑‑‑‑‑‑‑‑‑‑‑‑‑‑‑‑‑‑‑‑‑‑‑‑‑‑‑‑‑‑‑‑‑‑‑‑‑‑‑‑‑‑‑‑‑‑‑‑‑‑‑‑‑‑‑‑‑‑‑‑‑‑‑‑‑‑‑‑‑‑‑‑‑‑‑‑‑‑‑‑‑‑‑‑‑‑‑‑‑‑‑‑‑‑  >  NC_001604.1/8028‑8335`  
`‑‑‑‑‑‑‑‑‑‑‑‑‑‑‑‑‑‑‑‑‑‑‑‑‑‑‑‑‑‑‑‑‑‑‑‑‑‑‑‑‑‑‑‑‑‑‑‑‑‑‑‑‑‑‑‑‑‑‑‑‑‑‑‑‑‑‑‑‑‑‑‑‑‑‑‑‑‑‑‑‑‑‑‑‑‑‑‑‑‑‑‑‑‑‑‑‑‑‑‑‑‑‑‑‑‑‑‑‑‑‑‑‑‑‑‑‑‑‑‑‑‑‑‑‑‑‑‑‑‑‑‑‑‑‑‑‑‑‑‑‑‑‑‑‑‑‑‑‑‑‑‑‑‑‑‑‑‑‑‑‑‑‑‑‑‑‑‑‑‑‑‑‑‑‑‑‑‑‑‑‑‑‑‑‑‑‑‑‑‑‑‑‑‑‑‑‑‑‑‑‑‑‑‑‑‑‑‑‑‑‑‑‑‑‑‑‑‑‑‑‑‑‑‑‑‑‑‑‑‑‑‑‑‑‑‑‑‑‑‑‑‑‑‑‑‑‑‑‑‑‑‑‑‑‑‑‑‑‑‑‑‑‑‑‑‑‑‑‑‑‑‑‑‑‑‑‑‑‑‑‑‑‑‑‑‑‑‑‑‑‑‑‑‑‑‑‑‑‑‑gacaataaGAAGTTTTGGGCTACCGTAGAGTCCTCGGAGCATTCCTTCGAGGTTCCAATCTACGCTGAGACCCTAGACGAAGCTCTGGAGTTAGCCGAATGGCAATACGTTCCGGCTGGCTTTGAGGTTACTCGTGTGCGTCCTTGTGTAGCACCGAAGTAATACGACTCACTATTAGGGAAGACTCCCTCTGAGAAACCAAACGAAACCTAAAGGAGATTAACATTATGGCTAAGAAGATTTTCACCTCTGCGCTGGGTACCGCTGAACCTTACGCTTACATCGCCAAGCCGGACTACGGCAACGA  >  NC_001604.1/8939‑9237`  

                                                                                                                                                                                                                                                                                                                                                                                                                                                                                                                                                                                                                                 
`gagatgtgtataagagacagTTATCACCCGTGGTGACTTTGAGAAAGCAAAAGAAAAGAAACGTCCGGTTCTTAGTGTGGAAGTGACCCGCTTCCCGTTCGTCCGTCTGTTACTCAAACGAATCAAGGAGGTGTTCTGATGGGACTGTTAGATGGTGAAGCCTGGGAAAAAGAAAACCCGCCAGTACAAGCAACTGGGTGTATAGCTTGCTTAGAGAAAGATGACCGTTATCCACACACCTGTAACAAAGGAGCTAACGATATGACCGAACGTGAACAAGAGATGATCATTAAGTTGATAG                                                                                                                                                                                                                                                                                                                    <  1:148335/281‑1`  
`agagaccgGCGTCCACATCATTATCACCCGTGGTGACTTTGAGAAAGCAATAGACAAGAAACGTCCGGTTCTTAGTGTGGCAGTGATCCGCTTCCCGTTCGTCCGTCTGTTACTCAAACGAATCAAGGAGGTGTTCTGATGGGACTGTTAGATGGTGAAGCCTGGGAAAAAGAAAACCCGCCAGTACAAGCAACTGGGTGTATAGCTTGCTTAGAGAAAGATGACCGTTATCCACACACCTGTAACAAAGGAGCTAACGATATGACCGAACGTGAACAAGAGATGATCATTAAGTTGATAG                                                                                                                                                                                                                                                                                                                    <  1:171462/293‑1`  
`GGCACCGAGCGTCCACATCATTATCACCCGTGGTGACTTTGAGAAAGCAATAGACAAGAAACGTCCGGTTCTTAGTGTGGCAGTGACCCGCTTCCCGTTCGTCCGTCTGTTACTCAAACGAATCAAGGAGGTGTTCTGATGGGACTGTTAGATGGTGAAGCCTGGGAAAAAGAAAACCCGCCAGTACAAGCAACTGGGTGTATAGCTTGCTTAGAGAAAGATGACCGTTATCCACACACCTGTAACAAAGGAGCTAACGATATGACCGAACGTGAACAAGAGATGATCATTAAGTTGATAG                                                                                                                                                                                                                                                                                                                    >  1:26635/1‑301`  
`GGCACCGAGCGTCCACATCATTATCACCCGTGGTGACTTTGAGAAAGCAATAGACAAGAAACGTCCGGTTCTTAGTGTGGCAGTGACCCGCTTCCCGTTCGTCCGTCTGTTACTCAAACGAATCAAGGAGGTGTTCTGATGGGACTGTTAGATGGTGAAGCCTGGGAAAAAGAAAACCCGCCAGTACAAGCAACTGGGTGTATAGCTTGCTTAGAGAAAGATGACCGTTATCCACACACCGGTAACAAAGGAGCTAACGATATGAACGAACGTGAACAAGAGATGATCATTAAGTTGATAG                                                                                                                                                                                                                                                                                                                    >  1:234802/1‑301`  
`GGCACCCAGCGTCCACAGCATTAACACCCGGGGTGACTTTGAGAAAGCAAAAGACAAGAAACGTCCGGTTCTTAGTGGGGCAGGGACACGCTTCCCGTTCGTCCGTCTGTTACTCAAACGAATCAAGGAGGTGTTTTGATGGGACTGTTAGATGGTGAAGCCTGGGAAAAAGAAACCCCGCCAGTACAAGCAACTGGGTGTATAGCTTGCTTAGAGAAAGATGACCGTTATCCACACACCTGTAACAAAGGAGCTAACGATATGACCGAACGTGAACAAGAGATGATCATTAAGTTGATAG                                                                                                                                                                                                                                                                                                                    <  1:223410/301‑1`  
 `gaacggggctcCCACATCATTATCCCCCGTGGTGACTTGAGGAAAGCAATAGCCAAGAAACGTCCGGTTCTTAGTGTGGCAGTGACCCGCTTCCAGTTCGTCCGTCTGTTACTCAAACGAATCAAGGAGGTGTTCTGATGGGACTGTTAGATGGTGAAGCCTGGGAAAAAGAAAACCCGCCAGTACAAGCAACTGGGTGTATAGCTTGCTTAGAGAAAGATGACCGTTATCCACACACCTGTAACAAAGGAGCTAACGATATGACCGAACGTGAACAAGAGATGATCATTAAGTTGATAGA                                                                                                                                                                                                                                                                                                                   <  1:331119/290‑1`  
 `GCACCCAGCGTCCAAATCATTATCACCCGTGGTGACTGTGAGAAAGCGATAGACAAGAAACGTCCGGTTCTTAGTGTGGCAGTGACCCGCTTCCCGTTCGTCCGTCTGTTAATCAAACGAATCAAGGAGGTGTTCTGATGGGACTGTTGGATGGTGAAGCCTGGGAAAAAGAAAACCCGCCAGTACAAGCAACTGGGTGTATAGCTTGCTTAGAGAAAGATGACCGTTAACCACACACCTGTAACAAAGGAGCTAACGATATGACCGAACGTGAACAAGAGATGATCATTAAGTTGATAGA                                                                                                                                                                                                                                                                                                                   <  1:160804/301‑1`  
  `ccccgcaCGTCCACATCATTATCACCCGTTGTTACTTTGAGAAAGCACTAGCCAAGAACCGTCCGTTTCTTAGTTTGGCAGTGACCCCCTTCCCGTTCGTCCTTCTGTTACTCAACCGAATCAAGGAGTTGTTCTGATGGAGCTTTTAGATGGTGAAGCCTGGGAAAAAGAAACCCCGCCAGTACAAGCAACTGTGTGTATAGCTTGCTTAGAGAAAGATGACCGTTCTCCACACACCTGTAACAAAGGAGCTAACGATATGACCGAACGTGAACAAGAGATGATCATTAAGTTGATAGAC                                                                                                                                                                                                                                                                                                                  <  1:233821/294‑1`  
  `caccggggttcaACATCATTTTCACCCGTGGTGACTTTGAGAAAGCAATAGACAAGAAACGTCCGGTTCTTAGTGTGGCAGTGACCCGCTTCCCGTTCGTCCGTCTGTTACTCAAACGAATCAAGGAGGTGTTCTGATGGGACTGTTAGATGGTGAAGCCTGGGAAAAAGAAAACCCGCCAGTACAAGCAACTGGGTGTATAGCTTGCTTAGAGAAAGATGACCGTTATCCACACACCTGTAACAAAGGAGCTAACGATATGACCGAACGTGAACAAGAGATGATCATTAAGTTGATAGcg                                                                                                                                                                                                                                                                                                                  <  1:206286/289‑3`  
  `caaCGAGCGTCAAAATCATTATCACCCGTGGGGACTTTGGGAAAGCAATAGACAAGAAACTTCCGGTTCTTAGTGTGGCAGTGACCCGCTTACCGTTCGTCCGTCTGTTACTCAAACGAATCAAGGAGGTGTTCTGATGGGACTGTTAGATGGTGAAGCCTGGGAAAAAGAAAACCCGCCAGTACAAGCAACTGGGTGTATAGCTTGCTTAGAGAAAGATGACCGTTATCCACACACCTGTAACAAAGGAGCTAACGATATGACCGAACGTGAACAAGAGATGATCATTAAGTTGATAGAC                                                                                                                                                                                                                                                                                                                  <  1:24521/298‑1`  
  `atgtgtataagagaaagATTATCACCCGGGGTGACTTTGAGAAAGCAATAGACAAGAAACGTCCGGTTCTTAGTGGGGCAGTGACCCGCTTCCCGTTCGTCCGTCTGTTACTCAAACGAATCAAGGAGGTGTTCTGATGGGACTGTTAGATGGTGAAGCCTGGGAAAAAGAAAACCCGCCAGTACAAGCAACTGGGTGTATAGCTTGCTTAGAGAAAGATGACCGTTATCCACACACCTGTAACAAAGGAGCTAACGATATGACCGAACGTGAACAAGAGATGATCATTAAGTTGATAGAC                                                                                                                                                                                                                                                                                                                  <  1:116810/284‑1`  
  `agagacagGTCCACATCATTATCACCCGTGGTGACTTTGAGAAAGCAATAGACAAGAAACGTCCGGTTCTTAGTGTGGCAGTGACCCGCTTCCCGTTCGTCCGTCTGTTACTCAAACGAATCAAGGAGGTGTTCTGATGGGACTGTTAGATGGTGAAGCCTGGGAAAAAGAAAACCCGCCAGTACAAGCAACTGGTTGTATAGCTTGCTTAGAGAAAGATGACCGTTATCCACACACCTGTAACAAAGGAGCTAACGATATGACCGAACGTGAACAAGAGATGATCATTAAGTTGATAGAC                                                                                                                                                                                                                                                                                                                  <  1:66023/293‑1`  
  `aagagacaGTCAACATCATTATCACCCGGGGTGACTTTGAGAAAGCAATAGACAAGAAACGTCCGGTTCTTAGTGTGGCAGTGACCCGCTTCCCGTTCGTCCGTCTGTTACTCAAACGAATCAAGGAGGTGTTCTGATGGGACTGTTAGATGGTGAAGCCTGGGAAAAAGAAAACCCGCCAGTACAAGCAACTGGGTGTATAGCTTGCTTAGAGAAAGATGACCGTTATCCACACACCTGTAACAAAGGAGCTAACGATATGACCGAACGTGAACAAGAGATGATCATTAAGTTGATAGAC                                                                                                                                                                                                                                                                                                                  <  1:238187/293‑1`  
  `aACCGAGGTTCCAACTCATTAGCACCCGTGGTGACTTTGAGAAAGCAATAGACAAGAACCGTCCGGTTCTTAGTGTGGCAGGGACCCGCTTCCCGTTCGTCCGTCTGTTACTCAAACGAATCAAGGAGGTGTTCTGATGGGACTGTTAGATGGTGAAGCCTGGGAAAAAGAAACCCCGCCAGTACAAGCAACTGGGTGTATAGCTTGCTTAGAGAAAGATGACCGTTATCCACACACCTGTAACAAAGGAGCTAACGATATGACCGAACGTGAACAAGAGATGATCATTAAGTTGATAGAC                                                                                                                                                                                                                                                                                                                  <  1:154359/300‑1`  
  `aACCGAGCGTCCACATCATTATCACCCGTGGTGACGTTGAGAAAGCAATAGACAAGAAACGTCCGGTTCTTAGTGTGGCAGTGACCCGCTTCCCGTTCGTCCGTCTGTTACTCAAACGAATCAAGGAGGTGTTCTGATGGGACTGTTAGATGGTGAAGCCTGGGAAAAAGAAAACCCGCCAGTACAAGCAACTGGGTGTATAGCTTGCTTAGAGAAAGATGACCGTTATCCACACACCTGTAACAAAGGAGCTAACGATATGACCGAACGTGAACAAGAGATGATCATTAAGTTGATAGAC                                                                                                                                                                                                                                                                                                                  <  1:325558/300‑1`  
  `CACCGGCCGGCCCCATCATTATCCCCCGTGGTGCATTTGAGAAAGCAATAGACAAGAAACGTCCGGTTCTGAGTGTGGCAGTGACCCGCTTCCCGTTCGTCCGTCTGTTACTCAAACGAATCAAGGAGGTGTTCTGATGGTACTGTTAGATGGTGAGGCCTGGGAAAAAGAAAACCCGCCAGTACAAGCAACTGGGTGTATAGCTTTCTTAGAGAAAGATGACCGTTATCCACACACCTGTAACAAAGGAGCTAACGATATGACCGAACGTGAACAAGAGATGATCATTAAGTTGATAGAC                                                                                                                                                                                                                                                                                                                  <  1:277662/301‑1`  
  `CACCGATGGTCCACATCTTTATCCCCCATGGTGAGTATGAGAAAGCAAAAGGCAAGAAACGTCCGGTTCGTAGTGTGGCAGTGACCCGCTTCCCGTTCTTCCTTCTGTTACTCAAACGAATCAAGGAGGTGGTCTGAGGGGACTGTTAGATGGTGAAGCCTGGGAAAAAGACAACCCGCCAGTACAAGCAACTGGGTGTATAGCTTGCTTAGAGAAAGATGACCGTTATCCACACACCTGTAACAAAGGAGCTAACTATATGACCGAACGTGAACAAGAGATGATCATTAAGTTGATAGAC                                                                                                                                                                                                                                                                                                                  <  1:42589/301‑1`  
  `CACCGAGCGTCCACATCAGTATCACCCGTGGGGACTTTGAGAAAGCAATAGACAAGAAACGTCCGGTTCTGAGTGTGGCAGTGACCCGCTTCCCGTTCGTCCGTCTGTTACTCAAACGAATCAAGGAGGTGTTCTGATTGGACTGTTAGATGGTGAAGCCTGGGAAAAAGAAAACCCGCCAGTACAAGCAACTGGGTGTATAGCTTGCTTAGAGAAAGATGACCGTTATCCACACACCTGTAACAAAGGAGCTAACGATATGACCGAACGTGAACAAGAGATGATCATTAAGTTGATAGAC                                                                                                                                                                                                                                                                                                                  <  1:133237/301‑1`  
  `CACCGAGCGTCCAAATCATTATCACCCGTGGTGACTTTGAGAAAGCAATAGACAAGAAACTTCCGGTTCTTAGTGTGGCAGTGACCCGCTTCCCGTTCGTCCGTCTGTTACTCAAACGAATCAAGGAGGTGTTCTGATGGGACTGTTAGATGGTGAAGCCTGGGAAAAAGAAAACCCGCCAGTACAAGCAACTGGGTGTATAGCTTGCTTAGAGAAAGATGACCGTTATCCACACACCTGTAACAAAGGAGCTAACGATATGACCGAACGTGAACAAGAGATGATCATTAAGTTGATAGAC                                                                                                                                                                                                                                                                                                                  <  1:112240/301‑1`  
    `ataagggacagCATCATTATCACCCGTGGTGACTTTGAGAAAGCAATAGACAAGAAACGTCCGGTTCTTAGTGTGGCAGTGACCCGCTTCCCGTTCGTCCGTCTGTTACTCAAACGAATCAAGGAGGTGTTCTGATGGGACTGTTAGATGGTGAAGCCTGGGAAAAAGAAAACCCGCCAGTACAAGCAACTGGGTGTATAGCTTGCTTAGAGAAAGATGACCGTTATCCACACACCTGTAACAAAGGAGCTAACGATATGACCGAACGTGAACAAGAGATGATCATTAAGTTGATAGACAA                                                                                                                                                                                                                                                                                                                <  1:4335/290‑1`  
    `CCGAGCGTCCACATCATTATCACCCGTGGTGACTTTGAGAAAGCAATAGACAAGAAACGTCCGGTTCTTAGTGTGGCAGTGACCCGCTTCCCGTTCGTCCGTCTGTTACTCAAACGAATCAAGGAGGTGTTCTGATGGGACTGTTAGATGGTGAAGCCTGGGAAAAAGAAAACCCGCCAGTACAAGCAACTGGGTGTATAGCTTGCTTAGAGAAAGATGACCGTTATCCACACACCTGTAACAAAGGAGCTAACGATATGACCGAACGTGAACAAGAGATGATCATTAAGTTGATAGACAA                                                                                                                                                                                                                                                                                                                >  1:72686/1‑301`  
    `CCGAGCGTCCACATCATTATCACCCGTGGTGACTTTGAGAAAGCAATAGACAAGAAACGTCCGGTTCTTAGTGTGGCAGTGACCCGCTTCCCGTTCGTCCGTCTGTTACTCAAACGAATCAAGGAGGAGTTCTGATGGGACTGTTAGATGGTGAAGCCTGGGAAAAAGAAAACCCGCCAGTACAAGCAACTGGGTGTATAGCTTGCTTAGAGAAAGATGAACGTTATCCACACAGCTGTAACAAAGGAGCTAACGATATGACCGAACGTGAACAAGAGATGAACATTAAGTTGATAGACct                                                                                                                                                                                                                                                                                                                >  1:244537/1‑299`  
     `ttgtataagagacagTTATCACCCGTGGTGACTTTGAGAAAGCAATAGACAAGAAACGTCCGGTTCTTAGTGTGGCAGTGACCCGCTTCCCGTTCGTCCGTCTGTTACTCAAACGAATCAAGGAGGTGTTCTGATGGGACTGTTAGATGGTGAAGCCTGGGAAAAAGAAAACCCGCCAGTACAAGCAACTGGGTGTATAGCTTGCTTAGAGAAAGATGACCGTTATCCACACACCTGTAACAAAGGAGCTAACGATATGACCGAACGTGAACAAGAGATGATCATTAAGTTGATAGACAAT                                                                                                                                                                                                                                                                                                               <  1:202757/286‑1`  
     `gtgtataagagacagTTATCACCCGTGGTGACTTTGAGAAAGCAATAGACAAGAAACGTCCGGTTCTTAGTGTGGCAGTGACCCGCTTCCCGTTCGTCCGTCTGTTACTCAAACGAATCAAGGAGGTGTTCTGATGGGACTGTTAGATGGTGAAGCCTGGGAAAAAGAAAACCCGCCAGTACAAGCAACTGGGTGTATAGCTTGCTTAGAGAAAGATGACCGTTATCCACACACCTGTAACAAAGGAGCTAACGATATGACCGAACGTGAACAAGAGATGATCATTAAGTTGATAGACAAT                                                                                                                                                                                                                                                                                                               <  1:106885/286‑1`  
     `cggggtgCCACATCATTATCCCCCGTGGGGACTTTGAGAAAGCAATAGACAAGAAACGTCCGGTTCTTAGTGTGGCAGTGACCCGCTTCCCGTTCGTCCGTCTGTTACTCAAACGAATCAAGGAGGTGTTCTGAGGGGACTGTTAGATTGTGAAGCCTGGGAAAAAGAAAACCCGCCAGTACAAGCAACTGGGTGTATAGCTTGCTTAGAGAAAGATGACCGTTATCCACACACCTGTAACAAAGGAGCTAACGATATGACCGAACGTGAACAAGAGATGATCATTAAGTTGATAGACAAT                                                                                                                                                                                                                                                                                                               <  1:107400/294‑1`  
     `cggGCGTCAACATAATTATCACCCGTGGTGACTTTGAGAAAGCAATAGACAAGAAACGTCCGGTTCTTAGTGTGGCAGTGACCAGCTTCCCGTTCGTCCGTCTGTTACTCAAACGAATCAAGGAGGTGTTCTGATGGGACTGTTAGATGGTGAAGCCTGGGAAAAAGAAAACCCGCCAGTACAAGCAACTGGGTGTATAGCTTGCTTAGAGAAAGATGACCGTTATCCACACACCTGTAACAAAGGAGCTAACGAAATGACCGAACGTGAACAAGAGATGATCATTAAGTTGATAGACAAT                                                                                                                                                                                                                                                                                                               <  1:330233/298‑1`  
     `CGAGCGTCCACATCATTATCACCCTGGGTGACTTTGAGAAAGCAATAGACAAGAAACGTCCGGTTCTTAGTGTGGCAGTTACCCGCTTCCCGTTCGTCCGTCTGTTACTCAAACGAATCAAGGAGGTGTTCTGATGGGACTGTTAGATGGTGAAGCCTGGGAAAAAGAAAACCCGCCAGTACAAGCAACTGGGTGTATAGCTTGCTTAGAGAAAGATGACCGTTATCCACACACCTGTAACAAAGGAGCTAACGATATGACCGAACGTGAACAAGAGATGATCATTAAGTTGATAGACAAT                                                                                                                                                                                                                                                                                                               <  1:53021/301‑1`  
     `CGAGCGTCCACATCATTATCACCCGTGGTGACTATGAGACAGCAATAGACAAGAAACATCCGGTTCTTAGTGTGGAGTTGACCCGCGTCCAGTTCGTCCGTTTGTTGCTCAAACGAATGAAGGAGGTGTTCTGATGGGACTGTTAGAGGGTGAAGCCTGGGAAAAAGAAAACCCGCCAGTACAAGCAACTGGGTGTATAGCTTGATTAGAGAAAGATGACCGTTATCCACACACCTGTAACAAAGGAGCTAACGATATGACCGAACGTGAACAAGAGATGATCATTAAGTTGATAGACAAT                                                                                                                                                                                                                                                                                                               <  1:142112/301‑1`  
     `CGAGCGGCAAGATCATTATCACCCGGGGTGACTTTGAGAAAGAAAAAGACAAGAAACGACCGGTTCTGAGAGTGACAGTGACCGGCTGCCCGTTGGGCCGTCTGTGACTCAAAAGAAGCAAGGAGGTGTTCTGATGGGACTTTTAGATGGTGAAGCCTGGGAAAAAGAAAACCCGACAGTACAAGCAACTGGGTGTATAGCTTGCTTAGAGAAAGATGACCGTTATCCACACACCTGTAACAAAGGAGCTAACGATATGACCGAACGTGAACAAGAGATGATCATTAAGTTGATAGACAAT                                                                                                                                                                                                                                                                                                               <  1:79693/301‑1`  
      `GAGCGTCCACATCATTATCACCCGTGGTGACTTTGAGAAAGCAATAGACAAGAAACGTCCGGTTCTTAGTGTGGCAGTGACCCGCTTCCCGTTCGTCCGTCTGTTACTCAAACGAATCAAGGAGGTGTTCTGATGGGACTGTTAGATGGTGAAGCCTGGGAAAAAGAAAACCCGCCCGTACAAGCAACTGGGTGTATAGCTTGCTTAGCGAAAGATGACCGTTATCCACACACCTGTAACAAAGGAGCTAACGATATGACCGAACGTGAACAAGAGATGATCATTAAGTTGATAGACAATA                                                                                                                                                                                                                                                                                                              >  1:162334/1‑301`  
       `AGCGTCCACATCATTATCACCCGTGGTGACTTTGAGAAAGCAATAGACAAGAAACGTCCGGTTCTTAGTGTGGCAGTGACCCGCTTCCCGTTCGTCCGTCTGTTACTCAAACGAATCAAGGAGGTGTTCTGATGGGACTGTTAGATGGTGAAGCCTGGGAAAAAGAAAACCCGCCAGTACAAGCAACTGGGTGTATAGCTTGCTTAGAGAAAGATGACCGTTATCCACACACCTGTAACAAAGGAGCTAACGATATGACCGAACGTGAACAAGAGATGATCATTAAGTTGATAGACAATAA                                                                                                                                                                                                                                                                                                             >  1:90780/1‑301`  
       `AGCGTCCACATCATTATCACCCGTGGTGACTTTGAGAAAGCAATAGACAAGAAACGTCCGGTTCTTAGTGTGGCAGTGACCCGCTTCCCGTTCGTCCGTCTGTTACTCAAACGAATCAAGGAGGTGTTCTGATGGGACTGTTAGATGGTGAAGCCTGGGAAAAAGAAAACCCGCCAGTACAAGCAACTGGGTGTATAGCTTGCTTAGAGAAAGATGACCGTTATCCACACACCTGTAACAAAGGAGCTAACGATATGACCGAACGTGAACAAGAGATGATCATTAAGTTGATAGACAATAA                                                                                                                                                                                                                                                                                                             >  1:266707/1‑301`  
       `AGCGTCCACATCATTATCACCCGTGGTGACTTTGAGAAAGCAATAGACAAGAAACGTCCGGTTCTTAGTGTGGCAGTGACCCGCTTCCCGTTCGTCCGTCTGTTACTCAAACGAATCAAGGAGGTGTTCTGATGGGACTGTTAGATGGTGAAGCCTGGGAAAAAGAAAACCCGCCAGTACAAGCAACTGGGTGTATAGCTTGCTTAGAGAAAGATGACCGTTATCCACACACCTGTAACAAAGGAGCTAACGATATGACCGAACGTGAACAAGAGATGATCATTAAGTTGATAGACAATAA                                                                                                                                                                                                                                                                                                             >  1:268599/1‑301`  
       `AGCGTCCACATCATTATCACCCGTGGTGACTTTGAGAAAGCAATAGACAAGAAACGTCCGGTTCTTAGTGTGGCAGTGACCCGCTTCCCGTTCGTCCGTCTGTTACTCAAACGAATCAAGGAGGTGTTCTGATGGGACTGTTAGATGGTGAAGCCTGGGAAAAAGAAAACCCGCCAGTACAAGCAACTGGGTGTATAGCTTGCTTAGAGAAAGATGACCGTTAACCACACACCTGTAACAAAGGAGCTAACGATATGACCGAACGTGAACAAGAGATGATCATTAAGTTGATAGACAATAA                                                                                                                                                                                                                                                                                                             >  1:19544/1‑301`  
       `AGCGTCCACATCATTATCACCCGTGGTGACTTTGAGAAAGCAATAGACAAGAAACGTCCGGTTCTTAGTGTGGCAGTGACCCGCTTCCCGTTCGTCCGTCTGTTACTCAAACGAATCAAGGAGGTGTTCTGATGGGACTGTTAGATGGTGAAGCCTGGGAAAAAGAAAACACGCCAGTACAAGCAACTGGGTGTATAGCTTGCTTAGAGAAAGATGACCGTTATCCACACACCTGTAACAAAGGAGCTAACGATATGACCGAACGTGCACAAGAGATGATCATTAAGTTGATAGACAATAA                                                                                                                                                                                                                                                                                                             >  1:336723/1‑301`  
       `AGCGTCCACATCATTACCACCCGTGGTGACTTTGAGAAAGCAATAGACAAGAGACGTCCGGTTCTTAGTGTGGCAGTGACCCGCTTCCCGTTCGTCCGTCTGTTACTCAAACGAATCAAGGAGGTGTTCTGATGGGACTGTTAGATGGTGAAGCCTGGGAAAAATAAAACCCGCCAGTACAAGCAACTGGGTGTATAGCTTGCTTAGAGAAAGATGACCGTTATCCACACACCTGTAACAAAGGAGCTAACGATATGACCGAACGTGAACAAGAGATGATCATTAAGTTGATAGACAATAA                                                                                                                                                                                                                                                                                                             >  1:83692/1‑301`  
           `TCCACATCATTATCACCCGTGGTGACTTTGAGAAAGCAATAGACAAGAAACGTCCGGTTCTTAGTGTGGCAGTGACCCGCTTCCCGTTCGTCCGTCTGTTACTCAAACGAATCAAGGAGGTGTTCTGATGGGACTGTTAGATGGTGAAGCCTGGGAAAAAGAAAACCCGCCAGTACAAGCAACTGGGTGTATAGCTTGCTTAGAGAAAGATGACCGTTATCCACACACCTGTAACAAAGGAGCTAACGATATGACCGAACGTGAACAAGAGATGATCATTAAGTTGTTAGACAATAAttaa                                                                                                                                                                                                                                                                                                         >  1:268736/1‑297`  
           `TCCACATCATTATCACCCGTGGTGACTTTGAGAAAGCAATAGACAAGAAACGTCCGGTTCTTAGTGTGGCAGTGACCCGCTTCCCGTTCGTCCGTCTGTTACTCAAACGAATCAAGGAGGTGTTCTGATGGGACTGTTAGATGGTGAAGCCTGGGAAAAAGAAAACCCGCCAGTACAAGCAACTGGGTGTATAGCTTGCTTAGAGAAAGATGACCGTTATCCACACACCTGTAACAAAGGAGCTAACGATATGACCGAACGTGAACAAGAGATGATCATTAAGTTGATAGACtgtctctta                                                                                                                                                                                                                                                                                                         >  1:304702/1‑292`  
              `ACATCATTATCACCCGTGGTGACTTTGAGAAAGCAATAGACAAGAAACGTCCGGTTCTTAGTGTGGCAGTGACCCGCTTCCCGTTCGTCCGTCTGTTACTCAAACGAATCAAGGAGGTGTTCTGATGGGACTGTTAGATGGTGAAGCCTGGGAAAAAGAAAACCCGCCAGTACAAGCAACTGGGTGTATAGCTTGCTTAGAGAAAGATGACCGTTATCCACACACCTGTAACAAAGGAGCTAACGATATGACCGAACGTGAACAAGAGATGATCATTAAGTTGATAGACctgtctcttata                                                                                                                                                                                                                                                                                                      >  1:252244/1‑289`  
               `CATCATTATCACCCGTGGTTACTTTGAGAAAGCAATAGACAAGAAACGTCCGGTTCTTAGTGTGGCAGTGACCCGCTTCCCGTTCGTCCGTCTGTTACTCAAACGAATCAAGGAGGTGTTCTGATGGGACTGTTAGATGGTGAAGCCTGGGAAAGAGAAAACCCGCCAGTACAAGCAACTGGGTGTATAGCTTGCTTAGAGAAAGATGACGGTTATCCACACACCTGTAACAAAGGAGCTAACGATATTACCGAACGTGAACAAGAGATGAACATGAAGTTGATATACAttcatgactgtc                                                                                                                                                                                                                                                                                                     >  1:180010/1‑289`  
               `CATCATTATCACCCGTGGTGACTTTGAGAAAGCAATAGACAAGAAACGTCCGGTTCTTAGTGTGGCAGTGACCCGCTTCCCGTTCGTCCGTCTGTTACTCAAACGAATCAAGGAGGTGTTCTGATGGGACTGTTAGATGGTGAATCCTGGGAAAAAGAAAACCCGCCAGTACAAGCAACTGGGTGTATAGCTTGCTTAGAGAAAGATGACCGTTATCCACACACCTGTAACAAAGGAGCTAACGATATGACCGAACGTGAACAAGAGATGATCATTAAGTTGATAGACctgtctcttatac                                                                                                                                                                                                                                                                                                     >  1:145517/1‑288`  
               `CATCATTATCACCCGTGGTGACTTTGAGAAAGCAATAGACAAGAAACGTCCGGTTCTTAGTGTGGCAGTGACCCGCTTCCCGTTCGTCCGTCTGTTACTCAAACGAATCAAGGAGGTGTTCTGATGGGACTGTTAGATGGTGAAGCCTGGGAAAAAGAAAACCCGCCAGTACAAGCAACTGGGTGTATAGCTTGCTTAGAGAAAGATGACCGTTATCCACACACCTGTAACAACGTAGCTAACGATATGACCGAACGTGAACAAGAGATGATCATTAAGTTGATAGACAATActgactgtc                                                                                                                                                                                                                                                                                                     >  1:336389/1‑292`  
                 `TCATTATCACCCGTGGTGACTTTGAGAAAGCAATAGACAAGAAACGTCCGGTTCTTAGTGTGGCAGTGACCCGCTTCCCGTTCGTCCGTCTGTTACTCAAACGAATCAAGGAGGTGTTCTGATGGGACTGTTAGATGGTGAAGCCTGGGAAAAAGAAAACCCGCCAGTACAAGCAACTGGGTGTATAGCTTGCTTAGAGAAAGATGACCGTTATCCACACACCTGTAACAAAGGAGCTAACGATATAACCGAACGTGAACAAGAGATGATCATTAAGTTGATAGACctgtctcttatacac                                                                                                                                                                                                                                                                                                   >  1:161200/1‑286`  
                  `CATTATCACCCGTGGTGACTTTGAGAAAGCAATAGACAAGAAACGTCCGGATCTTAGTGTGGCAGTGACCCGCTTCCCGTTCGTCCGTCTGTTACTCAAACGAATCAAGGAGGTGTTCTGATGGGACTGTTAGATGGTGAAGCCTGGGAAAAAGAAAACCCGCCAGTACAAGCAACTGGGTGTATAGCTTGCTTAGAGAAAGATGACCGTTATCCACACACCTGTAACAAAGGAGCTAACGATATGACCGAACGTGAACAAGAGATGATCATTAAGTTGATAGctgtctcttatacacatc                                                                                                                                                                                                                                                                                                  >  1:298973/1‑283`  
                  `CATTATCACCCGTGGTGACGTTGAGCAAGCAATGGACAAGAAACGTCAGGTTCTTAGTGTGGCAGTGACCCGTAACACGTTCGTCCGTCTGTTACTCAAACGAATCAAGGAGGTGTTCTGATGGGACTGTTAGTAGGTGAAGCATGGGACAAAGAAAACCCGCCAGTACAAGCAACTGGGAGTATAGCTAGATTAGAGAAAGATGACCGTTGTCCACAAACCTGTAACAAAGGAGCTAACGATATGACCGCACGGATAAAAGAGATGATAAATAAGTTGATAGAAAATAAacaaagtcacc                                                                                                                                                                                                                                                                                                  >  1:110069/1‑290`  
                   `ATTATCACCCGTGGTGACTTTGAGAAAGCAATAGACAAGAAACGTCCGGTTCTTAGTGTGGCAGTGACCCGCTTCCCGTTCGTCCGTCTGTTACTCAAACGAATCAAGGAGGTGTTCTGATGGGACTGTTAGATGGTGAAGCCTGGGAAAAAGAAAACCCGCCAGTACAAGCAACTGGGTGTATAGCTTGCTTAGAGAAAGATGACCGTTATCCACACACCTGTAACAAAGGAGCTAACGATATGACCGAACGTGAACAAGAGATGATCATTAAGTTGATAGACctgtctcttatacacat                                                                                                                                                                                                                                                                                                 >  1:254555/1‑284`  
                    `TTATCACCCGTGGTGACTTTGAGAAAGCAATAGACAAGAAACGTCCGGTTCTTAGTGTGGCAGTGACCCGCTTCCCGTTCGTCCGTCTGTTACTCAAACGAATCAAGGAGGTGTTCTGATGGGACTGTTAGATGGTGAAGCCTGGGAAAAAGAAAACCCGCCAGTACAAGCAACTGGGTGTATAGCTTGCTTAGAGAAAGATGACCGTTATCCACACACCTGTACCAAAGGAGCTAACGATATGACCGACCGTGAACAAGAGATGATCATTAAGTTGATAGTCActctgtctcttctccac                                                                                                                                                                                                                                                                                                >  1:325150/1‑284`  
                    `TTATCACCCGTGGTGACTTTGAGAAAGCAATAGACAAGAAACGTCCGGTTCTTAGTGTGGCAGTGACCCGCTTCCCGTTCGTCCGTCTGTTACTCAAACGAATCAAGGAGGTGTTCTGATGGGACTGTTAGATGGTGAAGCCTGGGAAAAAGAAAACCCGCCAGTACAAGCAACTGGGTGTATAGCTTGCTTAGAGAAAGATGACCGTTATCCACACACCTGTAACAAAGGAGCTAACGATATGACCGAACGTGAACAAGAGATGATCATTAAGTTGATAGACctgtctcttatacacatc                                                                                                                                                                                                                                                                                                >  1:12431/1‑283`  
                       `TCACCCGTGGTGACTTTGAGAAAGCAATAGACAAGAAACGTCCGGTTCTTAGTGTGGCAGTGACCCGCTTCCCGGTCGTCCGTCTGTTACTCAAACGAATCAAGGAGGTGTTCTGATGGGACTGTTAGATGGTGAAGCCTGGGAAAAAGAAAACCCGCCAGTACAAGCAACTGTGTGTATAGCTTGCTTAGAGAAAGATGACCGTTATCCACACACCTGTAACAAAGGAGCTAACGATATGACCGAACGTGAACAAGAGATGATCATTAAGTTGATAGACAATAActgtctcttatacaca                                                                                                                                                                                                                                                                                             >  1:14264/1‑285`  
                        `CACCCGTGGTGACTTTGAGAAAGCAATAGACAAGAAACGTCCGGTTCTTAGTGTGGCAGTGACCCGCTTCCCGTTCGTCCGTCTGTTACTCAAACGAATCAAGGAGGTGTTCTGATGGGACTGTTAGATGGTGAAGCCTGGGAAAAAGAAAACCCGCCAGTACAAGCAACTGGGTGTATAGCTTGCTTAGAGAAAGATGACCGTTATCCACACACCTGTAACAAAGGAGCTAACGATATGACCGAACGTGAACAAGAGATGATCATTAGGTTGATAGctgtctcttatacacatctccgag                                                                                                                                                                                                                                                                                            >  1:10865/1‑277`  
                             `GTGGTGACTTTGAGAAAGCAATAGACAAGAAACGTCCGGTTCTTAGTGTGGCAGTGACCCGCTTCCCGTTCGTCCGTCTGTTACTCAAACGAATCAAGGAGGTGTTCTGATGGGACTGTTAGATGGTGACGCCTGGGACAAAGAAAACCCGCCAGTACAAGCAACTGGGTGTATAGCTTGCTTAGAGAAAGATGACCGTTATCCACACACCTGTAACAAAGGAGCTAACGATATGACCGAACGTGAACAAGAGATGATAATTAAGTTGATAGACctgtctattatacacatctccgagccc                                                                                                                                                                                                                                                                                       >  1:194247/1‑274`  
                                                           `acaGTCCGGTTCTTAGTGTGGCAGTGACCCGCGTCCCGTTCGTCAGTCTGTTACTCAAACGAATCAAGGAGGTGTTCAGATGGGACTGTTAGATGGTGAAGACTGGGAAAAAGAAACCCCGCCAGTACAAGCAACTGGGTGTATAGCTTGCTTAGATAAAGATGACCGTTATCCACACACCTGTAACAAAGGAGCTAACGATATGACCTAACGTGAACAAGAGATGATCATTAAGTTGATGGACAATAAGAAGTTTTGGGCTACCGTAGAGTCCTCGGAGCATTCCTTCGAGGTTCCAATC                                                                                                                                                                                                                                                         <  1:167211/298‑1`  
                                                                                                                                                    `TAGATGGTGAAGCCGGGAAAAAAGAAAACCCGCCAGTACAAGCAACTGGGTGTATAGCTTGCTTAGAGAAAGATGACCGTTATCCACACACCTGTAACAAAGGAGCTAACGATATGACCGAACGTGAACAAGAGATGATCATTAAGTTGATAGACAATAAGAAGTTTTGGGCTACCGTAGAGTCCTCGGAGCATTCCTTCGAGGTTCCAATCTACGCTGAGACCCTAGACAAAGCTCTGGAGTTAGCCGAATGGCAATACGTTCCGGCTGGCTTTGAGGTTACTCGTGTGCGTCCTTGTGT                                                                                                                                                                <  1:248006/301‑1`  
                                                                                                                                                                                 `CCGCCAGTACATGCAACTGTGTGTATAGCTTGCTTAGAGAAAGATGACCGTTATCCACACACCTGTAACAAAGGAGCTAACGATATGACCGAACGTGAACAAGAGATGATCATTAAGTTGATACACAATAAGAAGTTTTGGGCTACCGTAGAGTCCTCGGAGCATTCCTTCGAGGTTCCACTCTACGCTGAGACCCTAGACGAAGCTCTGGAGTTAGCCGAATGGCAATACGTTCCGGCTGGCTTTCAGGTTACTCGTGTGCGTCCTTGTGTAGCACCGAAGTAATACGACTCACTATTAG                                                                                                                                   <  1:126392/301‑1`  
                                                                                                                                                                                                                    `AGAGAAAGATGACCGTTATCCACACACCTGTAACAAAGGAGCTAACGATATGACCGAACGTGAACAAGAGATGATCATTAAGTTGATGGACAATAAGAAGTTTTGGGCTACCGTAGAGTCCTCGGAGCATTCCTTCGAGGTTCCAATCTACGCTGAGACCCTAGACGAAGCTCTGGAGTTAGCCGAATGGCAATACGTTCCGGCTGGCTTTGAGGTTACTAGTGTGCGTCCTTGTGTAGCACCGAAGTAATACGACTCACTATTAGGGAAGctgtctcttatacacatctccaagcccacg                                                                                                >  1:282101/1‑271`  
                                                                                                                                                                                                                                 `CGTTATCCACACACCTGTAACAAAGGAGCTAACGATATGACCGAACGTGAACAAGAGATGATCATTAAGTTGATAGACAATAAGAAGTTTTGGGCTACCGTAGAGTCCTCGGAGCATTCCTTCGAGGTTCCAATCTACGCTGAGACCCTAGACGAAGCTCTGGAGTTAGCCGAATGGCAATACGTTCCGGCTGGCTTTGAGGTTACTCGTGTGCGTCCTTGTGTAGCACCGAAGTAATACGACTCACTATTAGGGAAGACTCCCTCTGAGAAACCAAACGAAACCTAAAGGAGATTAACAT                                                                                   <  1:233016/301‑1`  
                                                                                                                                                                                                                                         `ACACACCTGTAACAAAGGAGCTAACGATATGACCGAACGTGAACAAGAGATGATCATTAAGTTGATAGACAATAAGAAGTTTTGGGCTACCGTAGAGTCCTCGGAGCATTCCTTCGAGGTTCCAATCTACGCTGAGACCCTAGACGAAGCTCTGGAGTTAGCCGAATGGCAATACGTTCCGGCTGGCTTTGAGGTTACTCGTGTGCGTCCTTGTGTAGCACCGAAGTAATACGACTGACTATTAGGGAAGACTCCCTCTGAGAAACCAAACGAAACCTAAAGGctgtctcttatacacatc                                                                           >  1:137399/1‑283`  
                                                                                                                                                                                                                                                                             `acagGAACAAGAGATGATCATTAAGTTGATAGAAAATAAGAAGTTTTGGGCTACCGTAGAGTCCTCGGAGCATTCCTTCGAGGTTCCAATCTACGCTGAGACCCTAGACGAAGCTCTGGAGTTAGCCGAATGGCAATACGTTCCGGCTGGCTTTGAGGTTACTCGTGTGCGTCCTTGTGTATCACCGAAGTAATACGACTCACTATTAGGGAAGACTCTCTCTGAGAAACCAAACGAAACCTAAAGGAGATTAACATTATGGCTAAGAAGATTTTCACCTCTGCGCTGGGTACCGCTGAAC                                       <  1:321178/297‑1`  
                                                                                                                                                                                                                                                                                    `ggaatgctccgaggactctacggtagcccaAAGAAGTTTTGGGCTACCGTAGAGTCCTCGGAGCATTCCTTCGAGGTTCCAATCTACGCTGAGACCCTAGACGAAGCTCTGGAGTTAGCCGAATGGCAATACGTTCCGGCTGGCTTTGAGGTTACTCGTGTGCGTCCTTGTGTAGCACCGAAGTAATACGACTCACTATTAGGGAAGTCTCCCTCTGAGAATCCAAACGAACCCTAAAGGAGATTAACATTATGGCTAAGAAGATTTTCACCTCTGCGCTGAGTACCGCTGAACCTTCCGC                                >  1:302430/31‑301`  
                                                                                                                                                                                                                                                                                                            `GACAATAAGAAGTTTTGGGCTACCGTAGAGTCCTCGGAGCATTCCTTCGAGGTTCCAATCTACGCTGAGACCCTAGACGAAGCTCTGGAGTTAGCCGAATGGCAATACGTTCCGGCTGGCTTTGAGGTTACTCGTGTGCGTCCTTGTGTAGCACCGAAGTAATACGACTCACTATTAGGGAAGACTCCCTCTGAGAAACCAAACGAAACCTAAAGGAGATTAACATTATGGCTAAGAAGATTTTCACCTCTGCGCTGGGTACCGCTGAACCTTACGCTTACATCGCCAAGCCGGACTACGG        <  1:232327/301‑1`  
                                                                                                                                                                                                                                                                                                            `GACAATAAGAAGTTTTGGGCTACCGTAGAGTACTCGGAGCATTCCTTCGAGGTTCCAATCTACGCTGAGACCCTAGACGAAGCTCTGGAGTTAGCCGAATGGCAATACGTTCCGGCTGGCTTTGAGGTTACTCGTGTGCGTCCTTGTGTAGCACCGAAGTAATACGACTCACTATTAGGGAAGACTCCCTCTGAGAAACCAAACGAAACCTAAAGGAGATTAACATTATGGCTAAGAAGATTTTCACCTCTGCGCTGGGTACCGCTGAACCTTACGCTTACATCGCCAAGCCGGACTACGG        >  1:303596/1‑301`  
                                                                                                                                                                                                                                                                                                            `GACAATAAGAAGTTTGGGGCTACCGTAGAGTCCTCGGAGCATTCCTTCGAGGTTCCAATCTACGCTGAGACCCTAGACGAAGCTCTGGAGTTAGCCGAATAGCAATACGTTCCGGCTGGCTTTGAGGTTACTCGTGTGCGTCCTTGTGTAGCACCGAAGTAATACGACTCACTATTAGGGAAGACTCCCTCTGAGAAACCAAACGAAACCTAAAGGAGATTAACATTATGGCTAAGAAGATTTTCACCTCTGCGCTGGGTACCGCTGAACCTTACGCTTACATCGCCAAGCCGGACTACGG        <  1:83399/301‑1`  
                                                                                                                                                                                                                                                                                                             `ACAATAAGAAGTTTTGGGCTACCGTAGAGTCCTCGGAGCATTCCTTCGAGGTTCCAATCTACGCTGAGACCCTAGACGAAGCTCTGGAGTTAGCCGAATGGCAATACGTTCCGGCTGGCTTTGAGGTTCCTCGTGTGCGTCCTTGTGTAGCACCGAAGTAATACGACTCACTATTAAGGAAGACTCCCTCTGAGAAACCCAACGAAACCTAAAGGAGATTAACATTATGGCTAAGAAGATTTTCACCTCTGCGCTGGGTACCGCTGAACCTTACGCTTATATCGCCAAGCCGGACTACGGC       >  1:334552/1‑301`  
                                                                                                                                                                                                                                                                                                             `ACAATAAGAAGTTTTGGGCTACCGTAGAGTCCTCGGAGCATTCCTTCGAGGTTCCAATCTACGCTGAGACCCTAGACGAAGCTCTGGAGTTAGCCGAATGGCAATACGTTCCGGCTGGCTTTGAGGTTACTCGTGTGCGTCCTTGTGTAGCACCGAAGTAATACGACTCACTATTAGGGAAGACTCCCTCTGAGAAACCAAACGAAACCTAACGGAGATTAACATTATGGCTAAGAAGATTTTCACCTCTGCGCTGGGTACCGCTGAACCTTACGCTTACATCGCCAAGCCGGACTGCGGC       <  1:9298/301‑1`  
                                                                                                                                                                                                                                                                                                             `ACAATAAGAAGTTTTGGGCTACCGTAGAGTCCTCGGAGCATTCCTTCGAGGTTCCAATCTACGCTGAGACCCTAGACGAAGCTCTGGAGTTAGCCGAATGGCAATACGTTCCGGCTGGCTTTGAGGTTACTCGTGTGCGTCCTTGTGTAGCACCGAAGTAATACGACTCACTATTAGGGAAGACTCCCTCTGAGAAACCAAACGAAACCTAAAGGAGATTAACATTATGGCTAAGAAGATTTTCACCTCTGCGCTGGGTACCGCTGAACCTTACGCTTACATCGCCAAGCCGGACTACGGC       <  1:49256/301‑1`  
                                                                                                                                                                                                                                                                                                             `ACAATAAGAAGTTTTGGGCTACCGTAGAGTCCTCGGAGCATTCCTTCGAGGTTCCAATCTACGCTGAGACCCTAGACGAAGCTCTGGAGTTAGCCGAATGGCAATACGTTCCGGCTGGCTTTGAGGTTACTCGTGTGCGTCCTTGTGTAGCACCGAAGTAATACGACTCACTATTAGGGAAGACTCCCTCTGAGAAACCAAACGAAACCTAAAGGAGATTAACATTATGGCTAAGAAGATTTTCACCTCTGCGCTGGGTACCGCTGAACCTTACGCTTACATCGCCAAGCCGGACTACGGC       <  1:57290/301‑1`  
                                                                                                                                                                                                                                                                                                             `ACAATAAGAAGTTTTGGGCTACAGTAGAGTCCTCGGAGCATTCCTTCGAGGTTCCAATCTACGCTGAGACCCTAGACGAAGCTCTGGAGTTAGCCGAATGGCAATACGTTCCGGCTGGCTTTGAGGTTACTCGTGTGCGTCCTTGTGTAGCACCGAAGTAATACGACTCACTATTAGGGAAGACTCCCTCTGAGAAACCAAACGAAACCTAAAGGAGATTAACATTATGGCTAAGAAGATTTTCACCTCTGCGCTGGGTACCGCTGAACCTTACGCTTACATCGCCAAGCCGGACTACGGC       <  1:342064/301‑1`  
                                                                                                                                                                                                                                                                                                             `ACAATAAGAAGTTTTGGGATACCGTAGAGTCCTCGGAGCATTCCTTCGAGGTTCCAAGCGACGCTGAGACCCTAGACGAAGCTAGGGAGTGAGCCGAAGGGCAAGACGTTCCGGCTGGCTTTGAGGTGACTAGTGTGAGTCCTTGTGTAGCACCGAAGTAATACGACTCACTATTAGGGAAGACTCCCTCTGAGAAACCAAACGAAACCTAAAGGAGATTAACATTATGGCTAAGAAGATTTTAACCTCTGCGCTGGGTACCGCTGAACCTTACGCTTACATCGCCAAGCCGGACTACGGC       <  1:47662/301‑1`  
                                                                                                                                                                                                                                                                                                              `CAATAAGACGTTTTGGGCTACCGTAGAGTCCTCGGAGCATTCCTTCGAGGTTCCAATCTACGCTGAGACCCTAGACGAAGCTCTGGAGTTAGCCGAATGGCAATACGTTCCGGCTGGCTTTGAGGTTACTCGTGTGCGTCCTTGTGTAGCACCGAAGTAATACGACTCACTATTAGGGAAGACTCCCTCTGAGAAACCAAACGAAACCTAAAGGAGATTAACATTATGGCTAAGAAGATTTTCACCTCTGCGCTGGGTACCGCTGAACCTTACGCTTACATCGCCAAGCCGGACTACGGCA      >  1:233729/1‑301`  
                                                                                                                                                                                                                                                                                                              `CAATAAGAAGTTTTGGGCTACCGTAGAGTCCTCGGAGCATTCCTTCGAGGTTCCAATCTACGCTGAGACCCTAGACGAAGCTCTGGAGTTAGCCGAATGGCAATACGTTCCGGCTGGCTTTGAGGTTACTCGTGTGCGTCCTTGTGTAGCACCGAAGTATTACGACTCACTATTAGGGAAGACTCCCTCTGAGAAACCAAACGAAACCTAAAGGAGATTAACATTATGGCTAAGAAGATTTTCACCTCTGCGCTGGGTACCGCTGTACCTTACGCTTACATCCCCAAGCCGGACTACGGCA      >  1:32362/1‑301`  
                                                                                                                                                                                                                                                                                                              `CAATAAGAAGTTTTGGGCTACCGTAGAGTCCTCGGAGCATTCCTTCGAGGTTCCAATCTACGCTGAGACCCTAGACGAAGCTCTGGAGTTAGCCGAATGGCAATACGTTCCGGCTGGCTTTGAGGTTACTCGTGTGCGTCCTTGTGTAGCACCGAAGTAATACGACTCACTATTAGGGAAGACTCCCTCTGAGAAACCAAACGAAACCTAAAGGAGATTAACATTATGGCTAAGAAGATTTTCACCTCTGCGCTGGGTACCGCTGAACCTTCCGCTTACATCGCCAAGCCGGACTACGGCA      >  1:60063/1‑301`  
                                                                                                                                                                                                                                                                                                               `AATAAGAAGTTTTGGGCTACCGTAGAGTCCTCGGAGCATTCCTCGGAGGTTCAAATCTACGCTGAGACCCTAGACGAAGCTCTGGAGTTAGACGAATGGCAATACGTTCCGGCTGGCTTTGAGGTTACTCGTGTGCGTCCTGGTGTAGCACCGAAGTAATACGACTCACTATTAGGGAAGACTCCCTCTGAGAAACCAAACGAAACCTAAGGGAGATTAACATTATGGCTAAGAAGATTTTCACCTCTGCGCTGGGTACCGCTGAACCTTACGCTTACATCGCCAAGCCGGACTATGGCAA     <  1:320661/301‑1`  
                                                                                                                                                                                                                                                                                                                `ATAAGAAGTTTTGGGCTACCGTAGAGTTCTCGGAGCATTCCTTCGAGGTTACAATCTACGCTGAGACCCTAGACGAAGCTCTGGAGTTAGCCGAATGGCAATACGTTCCGGCTGGCTTTGAGGTTAATCGTGTGCGTCCTTGTGTAGCACCGAAGTAATAAGACTCACTATTAGGGAAGACACCCTCTGAGAAACCAAACGAAACGTAAAGGAGATTAATAATATCGCTAAGAAGATTTTCAACTCTGCGTTGGGTAACACTGAACTTTAAGCTTACATCGCCAAGACGGACCACcgtaaa    >  1:299160/1‑295`  
                                                                                                                                                                                                                                                                                                                `ATAAGAAGTTTTGGGCTACCGTAGAGTCCTCGGATCATTCCTTCGAGGTTCCAATCTACGCTGAGCCCCTCTACTACGCTCTGGCGTTAGCCGAATGGCAATACGTTCCGGCTGGCTTTGAGGTTACTCGTGTGCGTCCTTGTGTAGCCCCGAAGTAATACTACTCACTATTAGGGAAGACTCCCTCTGCGCAACCAAACGAAACCTAAAGGAGATTAACATTATGGCTAATAAGATTTTCACCTCTGCGCTGGGTACCGCTGAACCTTACGCTTACATAGCCCAGCCGGACTCCGTCAAC    >  1:29458/1‑301`  
                                                                                                                                                                                                                                                                                                                `ATAAGAAGTTTTGGGCTACCGTAGAGTCCTCGGAGCATTCCTTCGAGGTTCCAATCTACGCTGAGACCCTAGACGAAGCTCTTGAGTTAGCCGAATGGCAATACGTTCCGGCTGGCTTTGAGGTTACTCGTGTGCGTCCTTGTGTAGCACCGAAGTAATACGACTCACTATTAGGGAAGACTCCCTCTGAGAAACCAAACGAAACCTAAAGGAGATTAACATTATTGCTAAGAAGATTTTCACCTCTGCGCTGGGTACCGCTGAACCTTACTCTTACATAGCCAAGCCGGACTACGGCAAa    >  1:150357/1‑300`  
                                                                                                                                                                                                                                                                                                                `ATAAGAAGTTTTGGGCTACCGTAGAGTCCTCGGAGCATTCCTTCGAGGTTCCAATCTACGCTGAGACCCTAGACGAAGCTCTGGAGTTAGCCGAATGGCAATACGTTCCGGCTGGCTTTGAGGTTCCTCGTGTGCGTCCTTGTGTAGCACCGAAGTAATACGACTCACTATTAGGGAAGACTCCCTCTGAGAAACCAAACGAAACCTAAAGGAGATTAACATTATGGCTAAGAAGATTTTCACCTCTGCGCTGGGTACCGCTGAACCTTACGCTTACCTCGCCAAGCCGGACTACGGCAAC    >  1:326436/1‑301`  
                                                                                                                                                                                                                                                                                                                `ATAAGAAGTTTTGGGCTACCGTAGAGTCCTCGGAGCATTCCTTCGAGGTTCCAATCTACGCTGAGACCCTAGACGAAGCTCTGGAGTTAGCCGAATGGCAATACGTTCCGGCTGGCTTTGAGGTTACTCGTGTGCGTCCTTGTGTAGCACCGAAGTAATCCGACTCACTATTAGGGAAGACTCCCTCTGAGAAACCAAACGAAACCTAAAGGAGATTAACATTATGGCTAAGAAGATTTTCACCTCTGCGCTGGGTACCGCTGCACCTTACGCTTACATCGCCAAGCCGGACTACGGCAAC    >  1:29453/1‑301`  
                                                                                                                                                                                                                                                                                                                `ATAAGAAGTTTTGGGCTACCGTAGAGTCCTCGGAGCATTCCTTCGAGGTTCCAATCTACGCTGAGACCCTAGACGAAGCTCTGGAGTTAGCCGAATGGCAATACGTTCCGGCTGGCTTTGAGGTTACTCGTGTGCGTCCTTGTGTAGCACCGAAGTAATACGACTCACTATTAGGGAAGACTCCCTCTGAGAAACCAAACGAAACCTAAAGGAGATTAACATTATGGCTAAGAAGATTTTCACCTCTGCGCTGGGTACCGCTGAACCTTACGCTTACATCGCCCAGCCGGACTACGGCAAC    >  1:234856/1‑301`  
                                                                                                                                                                                                                                                                                                                `ATAAGAAGTTTTGGGCTACCGTAGAGTCCTCGGAGCATTCCTTCGAGGTTCCAATCTACGCTGAGACCCTAGACGAAGCTCTGGAGTTAGCCGAATGGCAATACGTTCCGGCTGGCTTTGAGGTTACTCGTGTGCGTCCTTGTGTAGCACCGAAGTAATACGACTCACTATTAGGGAAGACTCCCTCTGAGAAACCAAACGAAACCTAAAGGAGATTAACATTATGGCTAAGAAGATTTTCACCTCTGCGCTGGGTACCGCTGAACCTTACGCTTACATCGCCAAGCCGGACTCCGGCAAa    >  1:35145/1‑300`  
                                                                                                                                                                                                                                                                                                                `ATAAGAAGTTTTGGGCTACCGTAGAGTCCTCGGAGCATTCCTTCGAGGTTCCAATCTACGCTGAGACCCTAGACGAAGCTCTGGAGTTAGCCGAATGGCAATACGTTCCGGCTGGCTTTGAGGTTACTCGTGTGCGTCCTTGTGTAGCACCGAAGTAATACGACTCACTATTAGGGAAGACTCCCTCTGAGAAACCAAACGAAACCTAAAGGAGATTAACATTATGGCTAAGAAGATTTTCACCTCTGCGCTGGGTACCGCTGAACCTTACGCTTACATCGCCAAGCCGGACTACGGCAAC    >  1:118210/1‑301`  
                                                                                                                                                                                                                                                                                                                `ATAAGAAGTTTTGGGCTACCGTAGAGTCCTCGGAGCATTCCTTCGAGGTTCCAATCTACGCTGAGACCCTAGACGAAGCTCTGGAGTTAGCCGAATGGCAATACGTTCCGGCTGGCTTTGAGGTTACTCGTGTGCGTCCTTGTGTAGCACCGAAGTAATACGACTCACTATTAGGGAAGACTCCCTCTGAGAAACCAAACGAAACCTAAAGGAGATTAACATTATGGCTAAGAAGATTTTCACCTCTGCGCTGGGTACCGCTGAACCTTACGCTTACATCGCCAAGCCGGACTACGGCAAC    >  1:199409/1‑301`  
                                                                                                                                                                                                                                                                                                                `ATAAGAAGTTTTGGGCTACCGTAGAGTCCTCGGAGCATTCCTTCGAGGTTCCAATCTACGCTGAGACCCTAGACGAAGCTCTGGAGTTAGCCGAATGGCAATACGTTCCGGCTGGCTTTGAGGTTACTCGTGTGCGTCCTTGTGTAGCACCGAAGTAATACGACTCACTATTAGGGAAGACTCCCTCTGAGAAACCAAACGAAACCTAAAGGAGATTAACATTATGGCTAAGAAGATTTTCACCTCTGCGCTGGGTACCGCTGAACCTTACGCTTACATCGCCAAGCCGGACTACGGCAAC    >  1:117147/1‑301`  
                                                                                                                                                                                                                                                                                                                `ATAAGAAGTTTTGGGCTACCGTAGAGTCCTCGGAGCATTCCTTCGAGGTTCCAATCTACGCTGAGACCCTAGACGAAGCTCTGGAGTTAGCCGAATGGCAATACGTTCCGGCTGGCTTTGAGGTTACTCGTGTGCGTCCTTGTGTAGCACCGAAGTAATACGACTCACTATTAGGGAAGACTCCCTCTGAGAAACCAAACGAAACCTAAAGGAGATTAACATTATGGCTAAGAAGATTTTCACCTCTGCGCTGGGTACCGCTGAACCTTACGCTTACATCGCCAAGCCGGACTACGGCAAC    <  1:34007/301‑1`  
                                                                                                                                                                                                                                                                                                                `ATAAGAAGTTTTGGGCTACCGTAGAGTCCTCGGAGCATTCCTTCGAGGTTCCAATCTACGCTGAGACCCTAGACGAAGCTCTGGAGTTAGCCGAATGGCAATACGTTCCGGCTGGCTTTGAGGTTACTCGTGTGCGTCCTTGTGTAGCACCGAAGTAATACGACTCACTATTAGGGAAGACTCCCTCTGAGAAACCAAACGAAACCTAAAGGAGATTAACATTAGGGCTAAGAAGATTTTCACCTATGCGCTGGGTACCGCTGAACCTTACGCATACATCGCCAAGCCGGAGTACGGCAAa    >  1:118217/1‑300`  
                                                                                                                                                                                                                                                                                                                  `AAGAAGTTTTGGGCTACCGTAGATTCATCGGAGCATTCCTTTGAGGTTCCAATCTACGGTGAGACCCTAGAGGAAGCTCTGGAGTTAGCCGAATGGCAATACGTTCCGGCTAGCTTTGAGGTTACTCGTGTGCGTCCTTGGGTAGCACCGAAGTAATACGACTCACTATTAGGGAAGACTCCCTCTGAGAAACCAAACGAAACCTAAAGGAGATTAACATTATGACTAAGAAGATTTTCACCTCTGCGCTGGGTACCGCTGAACCTTACGCTTACATCGCCAAGCCGGACTACGGCAACGA  <  1:159679/301‑1`  
                                                                                                                                                                                                                                                                                                                  `AAGAAGTTTTGGGCTACCGTAGAGTCCTCGGAGCATTCCTTCGATGTTCCAATCTACGCTGAGACCCTAGACGAAGCTCTGGAGTTAGCCGAATGGCAATACGTTCCGGCTGGCTTTGAGGTTACTCGTGTGCGTCCTTGTGTAGCACCGAAGTAATACGACTCACTATTAGGGAAGACTCCCTCTGAGAAACCAAACGAAACCTAAAGGATATTAACATTATGGCTAAGAAGATTTTCACCTCTGCGCTGGGTACCGCTGAACCTTACGCTTACATCGCCAAGCCGGACTACTGCAACGA  <  1:263012/301‑1`  
                                                                                                                                                                                                                                                                                                                  `AAGAAGTTTTGGGCTACCGTAGAGTCCTCGGAGCATTCCTTCGAGGTTCTAATCTACGCTGAGACCCTAGACGAAGCTCTGGAGTTAGCCGAATGGCAATACGTTCCGGCTGGCTTTGAGGTTACTCGTGTGCGTCCTTGTGTAGCACCGAAGTAATACGACTCACTATTAGGGAAGACTCCCTCTGAGAAACCAAACGAAACCTAACGGAGATTAACATTATGGCTAAGAAGATTTTCACCTCTGCGCTGGGTACCGCTGAACCTTACGCTTACATCGCCAAGCCGGACTACGGCAACGA  >  1:42603/1‑301`  
                                                                                                                                                                                                                                                                                                                  `AAGAAGTTTTGGGCTACCGTAGAGTCCTCGGAGCATTCCTTCGAGGTTCCAATCTACGCTGAGACCCTAGACGAAGCTCTGGAGTTAGCCGAATGGCAATACGTTCCGGCTGGCTTTGAGGTTACTCGTGTGCGTCCTTGTGTAGCACCGAAGTAATACGACTCACTATTAGTGAAGACTCCCTCTGAGAAACCAAACGAAACCTAAAGGAGATTAACATTATGGCTAAGAAGATTTTCACCTCTGCGCTGGGTAGCGCTGAACCTTACGCTTACATCGCCAAGCCGGACTACGGCAACGA  <  1:118172/301‑1`  
                                                                                                                                                                                                                                                                                                                  `AAGAAGTTTTGGGCTACCGTAGAGTCCTCGGAGCATTCCTTCGAGGTTCCAATCTACGCTGAGACCCTAGACGAAGCTCTGGAGTTAGCCGAATGGCAATACGTTCCGGCTGGCTTTGAGGTTACTCGTGTGCGTCCTTGTGTAGCACCGAAGTAATACGACTCACTATTAGGGAAGACTCCCTCTGAGAAACCAAACGAAACCTAAAGGAGATTAACATTATGGCTAAGAAGATTTTCACCTCTGCGCTGGGTACCGCTGAACCTTACGCTTACATCGCCAAGCCGGACTACGGCAACGA  <  1:344698/301‑1`  
                                                                                                                                                                                                                                                                                                                  `AAGAAGTTTTGGGCTACCGTAGAGTCCTCGGAGCATTCCTTCGAGGTTCCAATCTACGCTGAGACCCTAGACGAAGCTCTGGAGTTAGCCGAATGGCAATACGTTCCGGCTGGCTTTGAGGTTACTCGTGTGCGTCCTTGTGTAGCACCGAAGTAATACGACTCACTATTAGGGAAGACTCCCTCTGAGAAACCAAACGAAACCTAAAGGAGATTAACATTATGGCTAAGAAGATTTTCACCTCTGCGCTGGGTACCGCTGAACCTTACGCTTACATCGCCAAGCCGGACTACGGCAACGA  <  1:262452/301‑1`  
                                                                                                                                                                                                                                                                                                                  `AAGAAGTTTTGGGCTACCGTAGAGTCCTCGGAGCATTCCTTCGAGGTTCCAATCTACGCTGAGACCCTAGACGAAGCTCTGGAGTTAGCCGAATGGCAATACGTTCCGGCTGGCTTTGAGGTTACTCGTGTGCGTCCTTGTGTAGCACCGAAGTAATACGACTCACTATTAGGGAAGACTCCCTCTGAGAAACCAAACGAAACCTAAAGGAGATTAACATTATGGCTAAGAAGATTTTCACCTCTGCGCTGGGTACCGCTGAACCTTACGCTTACATCGCCAAGCCGGACTACGGCAACGA  <  1:32481/301‑1`  
                                                                                                                                                                                                                                                                                                                  `AAGAAGTTTTGGGCGACCGTAGAGTCCTCGGAGCATTCCTTCGAGGTTCCAATCTACGCTGAGACCCTAGACGAAGCTCTGGAGTTAGCCGAATGGCAATACGTTCCGGCTGGCTTTGAGGTTACTCGTGTGCGTCCTTGTGTAGCACCGAAGTAATACGACTCACTATTAGGGAAGACTCCCTCTGAGAAACCAAACGAAACCTAAAGGAGATTAACATTATGGCTAAGAAGATTTTCACCTCTGCGCTGGGTACCGCTGAACCTTACGCTTACATCGCCAAGCCGGACTACGGCAACGA  <  1:257198/301‑1`  

                                                                                                                                                                                                                                                                                                                                                                                                                                                                                                                                                                                                                                 
`GGCACCGAGCGTCCACATCATTATCACCCGTGGTGACTTTGAGAAAGCAATAGACAAGAAACGTCCGGTTCTTAGTGTGGCAGTGACCCGCTTCCCGTTCGTCCGTCTGTTACTCAAACGAATCAAGGAGGTGTTCTGATGGGACTGTTAGATGGTGAAGCCTGGGAAAAAGAAAACCCGCCAGTACAAGCAACTGGGTGTATAGCTTGCTTAGAGAAAGATGACCGTTATCCACACACCTGTAACAAAGGAGCTAACGATATGACCGAACGTGAACAAGAGATGATCATTAAGTTGATAGACAATAA‑‑‑‑‑‑‑‑‑‑‑‑‑‑‑‑‑‑‑‑‑‑‑‑‑‑‑‑‑‑‑‑‑‑‑‑‑‑‑‑‑‑‑‑‑‑‑‑‑‑‑‑‑‑‑‑‑‑‑‑‑‑‑‑‑‑‑‑‑‑‑‑‑‑‑‑‑‑‑‑‑‑‑‑‑‑‑‑‑‑‑‑‑‑‑‑‑‑‑‑‑‑‑‑‑‑‑‑‑‑‑‑‑‑‑‑‑‑‑‑‑‑‑‑‑‑‑‑‑‑‑‑‑‑‑‑‑‑‑‑‑‑‑‑‑‑‑‑‑‑‑‑‑‑‑‑‑‑‑‑‑‑‑‑‑‑‑‑‑‑‑‑‑‑‑‑‑‑‑‑‑‑‑‑‑‑‑‑‑‑‑‑‑‑‑‑‑‑‑‑‑‑‑‑‑‑‑‑‑‑‑‑‑‑‑‑‑‑‑‑‑‑‑‑‑‑‑‑‑‑‑‑‑‑‑‑‑‑‑‑‑‑‑‑‑‑‑‑‑‑‑‑‑‑‑‑‑‑‑‑‑‑‑‑‑‑‑‑‑‑‑‑‑‑‑‑‑‑‑‑‑‑‑‑‑‑‑‑‑‑‑‑‑‑‑‑‑‑‑  >  NC_001604.1/8028‑8335`  
`‑‑‑‑‑‑‑‑‑‑‑‑‑‑‑‑‑‑‑‑‑‑‑‑‑‑‑‑‑‑‑‑‑‑‑‑‑‑‑‑‑‑‑‑‑‑‑‑‑‑‑‑‑‑‑‑‑‑‑‑‑‑‑‑‑‑‑‑‑‑‑‑‑‑‑‑‑‑‑‑‑‑‑‑‑‑‑‑‑‑‑‑‑‑‑‑‑‑‑‑‑‑‑‑‑‑‑‑‑‑‑‑‑‑‑‑‑‑‑‑‑‑‑‑‑‑‑‑‑‑‑‑‑‑‑‑‑‑‑‑‑‑‑‑‑‑‑‑‑‑‑‑‑‑‑‑‑‑‑‑‑‑‑‑‑‑‑‑‑‑‑‑‑‑‑‑‑‑‑‑‑‑‑‑‑‑‑‑‑‑‑‑‑‑‑‑‑‑‑‑‑‑‑‑‑‑‑‑‑‑‑‑‑‑‑‑‑‑‑‑‑‑‑‑‑‑‑‑‑‑‑‑‑‑‑‑‑‑‑‑‑‑‑‑‑‑‑‑‑‑‑‑‑‑‑‑‑‑‑‑‑‑‑‑‑‑‑‑‑‑‑‑‑‑‑‑‑‑‑‑‑‑‑‑‑‑‑‑‑‑‑‑‑‑‑‑‑‑‑‑gacaataaGAAGTTTTGGGCTACCGTAGAGTCCTCGGAGCATTCCTTCGAGGTTCCAATCTACGCTGAGACCCTAGACGAAGCTCTGGAGTTAGCCGAATGGCAATACGTTCCGGCTGGCTTTGAGGTTACTCGTGTGCGTCCTTGTGTAGCACCGAAGTAATACGACTCACTATTAGGGAAGACTCCCTCTGAGAAACCAAACGAAACCTAAAGGAGATTAACATTATGGCTAAGAAGATTTTCACCTCTGCGCTGGGTACCGCTGAACCTTACGCTTACATCGCCAAGCCGGACTACGGCAACGA  >  NC_001604.1/8939‑9237`

  

Alignment Legend

Aligned base mismatch/match (shaded by quality score): `ATCG/ATCG` `< 3 ≤` `ATCG/ATCG` `< 10 ≤` `ATCG/ATCG` `< 20 ≤` `ATCG/ATCG` `< 34 ≤` `ATCG/ATCG` `< 38 ≤` `ATCG/ATCG`

Unaligned base: `atcg`    Masked matching base: `atcg`    Alignment gap: `‑`    Deleted base: `‑`

Reads not counted as support for junction

read\_name Not counted due to insufficient overlap past the breakpoint.

read\_name Not counted due to not crossing MOB target site duplication.
